# Integration of Boruta algorithm and latent class analysis for risk factors of 30-day mortality in pediatric hemophagocytic lymphohistiocytosis based on peripheral blood indicators

**DOI:** 10.3389/fped.2026.1803651

**Published:** 2026-05-22

**Authors:** Chuncan Wu, Chuan Tian, Huidie Liang, Lili Liu, Zhonglv Ye, Weijun Huang, Xiaohuan Mo, Qiping Yang, Xiang Lan

**Affiliations:** Department of Pediatrics, Affiliated Hospital of Guangdong Medical University, Zhanjiang, China

**Keywords:** 30-day mortality risk, boruta algorithm, children, hemophagocytic lymphohistiocytosis, latent class analysis, prediction model

## Abstract

**Objective:**

To screen key peripheral blood indicators based on the Boruta feature selection algorithm, construct a latent class analysis (LCA) model, identify clinically heterogeneous subtypes of pediatric hemophagocytic lymphohistiocytosis (HLH), and evaluate the 30-day mortality risk following diagnosis.

**Methods:**

A retrospective cohort study design was employed, enrolling 133 children diagnosed with HLH at the Children's Medical Center of the Affiliated Hospital of Guangdong Medical University between January 1, 2015, and December 30, 2024. Demographic characteristics and laboratory indicators at admission were collected. Patients were categorized into a non-survivor group (29 cases) and a survivor group (104 cases) based on their 30-day survival outcomes after diagnosis. Univariate logistic regression analysis was initially conducted for preliminary variable screening, followed by the Boruta algorithm to eliminate feature noise and identify key predictors. An LCA model was constructed based on the screened variables for subgroup classification, and model fit was evaluated using Akaike information criterion (AIC), Bayesian information criterion (BIC), and entropy, among other metrics. Additionally, Shapley Additive exPlanations (SHAP) analysis, importance scoring, and restricted cubic spline (RCS) models were employed to interpret key variables and explore nonlinear relationships.

**Results:**

After dual screening through univariate analysis and the Boruta algorithm, six key predictive variables were identified: activated partial thromboplastin time (APTT), mean corpuscular hemoglobin (MCH), central nervous system (CNS) involvement, cyclosporine treatment, C-reactive protein (CRP), and imaging-confirmed hepatomegaly. LCA modeling demonstrated that the 2-class model exhibited the best fit (AIC = 1,507.750, BIC = 1,573.030, Entropy = 0.702), stratifying patients into a low-risk group (72%) and a high-risk group (28%). Survival analysis revealed a significantly lower 30-day cumulative survival rate in the high-risk group compared to the low-risk group (51.40% vs. 88.50%, Log-rank *P* < 0.001). Notably, patients in the high-risk group faced a 7.42-fold increased risk of 30-day mortality (*HR* = 7.4184, 95% CI: 2.6274–20.9205, *P* < 0.001). SHAP analysis indicated that APTT contributed most to the prediction, while RCS analysis revealed a monotonically increasing relationship between APTT and mortality risk, with elevated MCH levels demonstrating a significant protective trend.

**Conclusion:**

The analytical strategy integrating the Boruta algorithm and LCA effectively identifies clinical subtypes of pediatric HLH with distinct mortality risk trajectories based on routine peripheral blood indicators.

## 1 Introduction

Pediatric hemophagocytic lymphohistiocytosis (HLH) is an immune dysregulation syndrome triggered by genetic defects, infections, malignancies, or autoimmune diseases. Its core pathological mechanism involves dysfunction of cytotoxic T lymphocytes and natural killer (NK) cells, leading to excessive macrophage activation and secretion of large amounts of pro-inflammatory cytokines, subsequently inducing cytokine storm and systemic multiple organ dysfunction syndrome (1, 2). With the promotion of the HLH-2004 immunochemotherapy protocol and the maturation of hematopoietic stem cell transplantation (HSCT) technology, the 5-year overall survival rate of pediatric patients has reached approximately 62% (3). However, this improvement in long-term prognosis has not completely eliminated the perilous characteristics during the acute phase of the disease. Studies indicate that approximately 20%–25% of pediatric patients still die during the early stage after diagnosis or before HSCT (4). Within the first 30 days following diagnosis, patients often face extremely high mortality risk due to severe coagulation dysfunction, central nervous system (CNS) involvement, or refractory infections (5).

Clinical practice demonstrates that pediatric HLH exhibits high phenotypic heterogeneity, with substantial variations among different patients in disease onset rapidity, degree of organ involvement, and response to initial treatment. Traditional prognostic assessment systems predominantly rely on linear thresholds of single biomarkers (such as ferritin, fibrinogen, soluble CD25, etc.) or conventional regression models (6). These approaches assume that risk factors exert homogeneous linear effects across the entire population, making it difficult to capture complex systemic inflammatory network fluctuations and nonlinear interactions among variables. In recent years, advanced machine learning and statistical clustering algorithms have demonstrated immense potential in dissecting complex clinical syndromes, offering novel perspectives to overcome the limitations of traditional linear models. In the prognostic modeling of severe COVID-19, the Boruta algorithm has been successfully utilized to effectively filter out high-dimensional noise and precisely identify non-linear core inflammatory biomarkers that are frequently overlooked by conventional regression methods (7). Similarly, Latent Class Analysis (LCA) has driven a methodological paradigm shift in understanding highly heterogeneous clinical syndromes. A landmark study by Seymour et al. utilized LCA to conduct deep feature mining within a highly complex sepsis cohort, successfully deriving four distinct clinical phenotypes. Crucially, they isolated a high-risk phenotype characterized by high mortality and early multi-organ failure, which conventional severity scores failed to identify independently, thereby providing a critical foundation for individualized intervention in critically ill patients (8). Despite the significant success of these advanced algorithms in other hyperinflammatory syndromes, their application in the prognostic assessment of hemophagocytic lymphohistiocytosis (HLH) remains limited, with most preliminary attempts restricted to single algorithms. While existing studies have identified blood urea nitrogen, D-dimer, and other indicators as effective predictors for adult HLH through single machine learning algorithms (9), substantial differences exist between children and adults regarding etiological spectrum, immune response patterns, and physiological compensatory reserve functions. Consequently, directly extrapolating adult prediction models or relying on isolated indicators presents significant limitations in applicability for early risk stratification in pediatric populations (10). Therefore, constructing an analytical strategy capable of deeply integrating peripheral blood indicators and identifying potentially heterogeneous subtypes within the population holds important clinical significance for elucidating specific risk patterns of early mortality in pediatric HLH.

To overcome the limitations of traditional linear models in resolving individual heterogeneity, latent class analysis (LCA), as a person-centered probabilistic statistical modeling technique, provides a novel methodological perspective for precise classification of pediatric HLH (11). Unlike regression analysis that focuses on linear relationships between variables, LCA aims to derive latent categorical variables using observable indicators, thereby partitioning heterogeneous populations into subgroups with distinct characteristics. To enhance the model's dimensionality reduction efficiency and clinical interpretability, this study introduced the Boruta feature selection algorithm as a pre-screening tool. Based on the random forest framework, this algorithm constructs shadow features and compares their importance scores, effectively stripping redundant noise from routine blood count and biochemical indicators to precisely identify core predictors significantly associated with outcomes (12). Based on this, the present study aimed to construct and validate an LCA prediction model for 30-day mortality risk in pediatric HLH using key peripheral blood indicators screened through the Boruta algorithm, with the objectives of identifying clinical subtypes with different prognostic trajectories through analysis of specific combinatorial patterns among indicators, quantitatively evaluating mortality risk probability within 30 days after diagnosis for each latent class, and providing evidence-based decision support for early clinical intervention.

## 2 Materials and methods

### 2.1 Study population

This study employed a retrospective cohort design. Children diagnosed with HLH who were hospitalized at the Children's Medical Center of the Affiliated Hospital of Guangdong Medical University between January 1, 2015, and December 30, 2024, were selected as study subjects. For children with multiple hospitalizations, only data from the first admission were included.

Inclusion criteria: (1) age <18 years; (2) diagnostic criteria: strict adherence to the HLH-2004 diagnostic guidelines published by the Histiocyte Society (13), whereby patients must fulfill molecular biological diagnosis (presence of known HLH-related gene mutations) or meet at least 5 of the following 8 clinical and laboratory criteria: ① fever; ② splenomegaly; ③ cytopenias (affecting two or three lineages in peripheral blood); ④ hypertriglyceridemia and/or hypofibrinogenemia; ⑤ hemophagocytosis identified in bone marrow, spleen, or lymph nodes; ⑥ low or absent NK cell activity; ⑦serum ferritin (SF) ≥500 μg/L; ⑧elevated soluble CD25 (sCD25) level (≥2,400 U/mL).

Exclusion criteria: (1) prior intervention interference: patients who had undergone HSCT treatment before enrollment, as their immune baseline has fundamentally altered; (2) missing key outcomes: patients lost to follow-up after discharge or whose medical records could not trace survival outcomes within 30 days after diagnosis; (3) Severe data deficiency: core peripheral blood indicators (such as complete blood count, coagulation function, etc.) with missing rates exceeding 20% that cannot be effectively repaired through imputation techniques.

This study strictly adhered to the ethical principles of the Declaration of Helsinki and its revised versions. The research protocol was submitted to and approved by the Medical Ethics Committee of the Affiliated Hospital of Guangdong Medical University (Ethics Approval No. PJKT2026-029). Given that this study is a retrospective observational study that only collects and analyzes existing clinical medical records without intervening in patient treatment plans, and all personal privacy information of subjects has been strictly de-identified during the research process with controllable risks, the Ethics Committee approved a waiver of informed consent after deliberation.

### 2.2 Outcome definition and grouping

The primary endpoint of this study was defined as all-cause mortality within 30 days from the date of HLH diagnosis. Information on patient survival status was obtained through review of the hospital electronic medical record system combined with telephone follow-up, with the follow-up cutoff date being 30 days after diagnosis or the date of death occurrence. Based on 30-day survival outcomes, the study cohort was divided into a non-survivor group and a survivor group. To ensure accuracy of the prediction model and exclude interference of treatment interventions on biomarkers, all laboratory indicators included in the analysis (including complete blood count, coagulation function, and biochemical indicators) were strictly limited to baseline data collected on the day of diagnosis or within 24 h before diagnosis, and all samples were collected before patients received HLH-specific induction therapy (such as dexamethasone, etoposide, or cyclosporine).

### 2.3 Laboratory indicator detection

The collection time window for all laboratory indicators included in the analysis was strictly limited to the day of HLH diagnosis or within 24 h before diagnosis. To ensure homogeneity and accuracy of test data, blood sample collection was performed by professional nursing staff who received standardized training. If multiple test records existed within the specified time window, the value closest to the HLH diagnosis time point and obtained before specific induction therapy was selected as baseline data for analysis to objectively reflect the initial immune-inflammatory state of patients. All samples were processed strictly according to clinical laboratory standard operating procedures. Complete blood count samples were tested using an automated hematology analyzer (Sysmex XN-3000 series); biochemical, coagulation, and immune indicators were completed using corresponding supporting automated biochemical/immune analysis systems. This study focused on extracting and recording immune-inflammatory and organ function indicators: C-reactive protein (CRP), procalcitonin (PCT), NK cell activity, alanine aminotransferase (ALT), aspartate aminotransferase (AST), lactate dehydrogenase (LDH), total protein (TP), and serum ferritin (SF). Blood cell count indicators: white blood cell count (WBC), absolute neutrophil count (ANC), lymphocyte count (LYM), monocytes, red blood cell count (RBC), hemoglobin (Hb), mean corpuscular hemoglobin (MCH), mean corpuscular hemoglobin concentration (MCHC), red cell distribution width (RDW), platelet count (PLT), and reticulocytes. Coagulation function indicators: fibrinogen (FIB) and activated partial thromboplastin time (APTT).

### 2.4 Variable screening

Univariate preliminary screening: Variables with potential prognostic relevance were first explored using univariate logistic regression; however, final feature selection relied on the Boruta algorithm to minimize bias introduced by isolated statistical thresholds. Feature importance screening: The Boruta algorithm was further employed to assess and screen the importance of the above candidate features. For the Boruta feature selection algorithm, the underlying Random Forest model was executed with the default configuration of 500 trees. To ensure rigorous convergence, the iteration termination rule was set to a maximum of 500 runs (max Runs = 500), and the confidence level (*P*-value threshold) for comparing the *Z*-scores of original features with shadow features was strictly set at 0.01. Furthermore, any features remaining as Tentative after the maximum iterations were conclusively resolved into Confirmed or Rejected decisions using a tentative rough fix function based on their median *Z*-scores. Considering the predictive contribution of features comprehensively, 6 key variables were ultimately retained. Multicollinearity assessment: Generalized variance inflation factors (GVIF) were used to assess multicollinearity among variables (excluding variables with GVIF >5), which were deemed suitable for subsequent LCA modeling.

### 2.5 Sample size calculation

Sample size estimation in this study was based on the incidence estimation formula for cohort studies. Referring to relevant literature and clinical data, the short-term mortality rate of pediatric patients was assumed to be approximately 20% (*P* = 0.20). To ensure statistical power, confidence level was set at 95% $\left(\alpha =0.05,Z_{1-\alpha /2}=1.96\right)$. Considering that this study is a retrospective analysis, the allowable error was set at 7.5%. The pmsampsize package in *R* was used to assist calculation, with the sample size calculation formula as follows: $N=\frac{Z^{2}\times P(1-P)}{\delta^{2}}=\frac{1.96^{2}\times 0.20\times (1-0.20)}{0.075^{2}}\approx 109$. Based on the above formula, the initial sample size was calculated to be 109 cases. Considering possible information loss or matching attrition during retrospective data collection, a 10% sample attrition rate was set $N_{\mathrm{final}}=\frac{N}{1-0.10}=\frac{109}{0.9}\approx 121$. The final estimated required sample size was approximately 121 cases. This study ultimately included 133 children who met the screening criteria, with the sample size exceeding the theoretical estimate and satisfying the statistical requirements of the study.

In addition to the incidence-based estimation, the specific sample size required for LCA was mathematically determined based on the minimum required size for the smallest latent class. According to standard structural equation modeling guidelines (the *N*:*q* ratio) (14), stable parameter estimation requires at least 5 observations per freely estimated parameter within a specific class. For our intended model comprising 6 features (3 categorical and 3 continuous indicators), the algorithm estimates exactly 6 specific parameters per latent class (3 conditional probabilities and 3 conditional means). Therefore, the absolute minimum sample size required for the smallest subgroup (the high-risk class) is calculated as *N* class_min = 6 × 5 = 30 patients. Based on the epidemiological literature cited in our introduction, the early high-risk/mortality phenotype accounts for approximately 25% of the pediatric HLH population. To statistically capture a minimum of 30 high-risk patients with a prevalence of 25%, the total required sample size is geometrically calculated as *N* total_min = 30/0.25 = 120. Our actual enrolled cohort of 133 patients precisely exceeds this LCA-derived mathematical threshold of 120, thereby rigorously justifying the adequacy of the sample size for valid mixture modeling.

### 2.6 Statistical analysis

All statistical analyses were completed using R version 4.4.2 software. Before analysis, raw data were cleaned and quality-controlled to ensure data integrity. For baseline data, normally distributed continuous variables were expressed as mean standard deviation $\left(\bar{x}\pm s\right)$, with between-group comparisons using independent samples *t*-test; non-normally distributed continuous variables were presented as median and interquartile range (*M* [*P25*, *P75*]), with between-group comparisons using Mann–Whitney *U* test; categorical variables were expressed as frequency and percentage [*n*(%)] with between-group comparisons using chi-square test or Fisher's exact test.

This study utilized univariate logistic regression analysis for preliminary screening of potential prognostic variables (*P* < 0.05), followed by introduction of the Boruta algorithm, which eliminated noise by comparing *Z*-scores of original features with their shadow features to identify key predictive features. Finally, GVIF of retained variables were calculated to exclude multicollinearity among variables (GVIF < 5). LCA models were constructed based on screened key variables. Model fit evaluation metrics included AIC, BIC, adjusted BIC (aBIC), log-likelihood, entropy, Lo-Mendell-Rubin likelihood ratio test (LMR), and bootstrap likelihood ratio test (BLRT). When determining the optimal number of latent classes, the Bayesian Information Criterion (BIC) was established as the primary prioritized criterion, as it rigorously penalizes model complexity and is widely recognized as the most robust metric for class enumeration, particularly for our specific sample size. Other indices were utilized as secondary reference criteria: minimum AIC and aBIC, entropy >0.70 (indicating adequate classification quality), and statistically significant results for both LMR and BLRT tests (*P* < 0.05). To evaluate the prognostic significance of the identified latent classes, Kaplan–Meier survival curves were constructed and compared using the log-rank test. Furthermore, Cox proportional hazards regression models were employed to calculate hazard ratios (HR) and their 95% confidence intervals (CI).To flexibly model and visualize the potential non-linear dose-response relationships between continuous variables and mortality risk, restricted cubic spline (RCS) analysis was performed using the plotRCS package with its default optimal knot configurations. Furthermore, instead of relying on arbitrary visual estimations, piecewise linear regression (segmented regression) was applied using the segmented package to statistically determine the precise structural breakpoints. These mathematically derived breakpoints were subsequently defined as the exact clinical cut-off values and reference points [odds ratio (OR) = 1.0] in the RCS models.

Additionally, *E*-values were calculated for sensitivity analysis to evaluate result robustness, and Shapley Additive exPlanations (SHAP) methods and variable importance scores were introduced for model visualization and interpretation, quantifying the contribution of each feature to individual prediction results. Two-sided *P* < 0.05 was considered statistically significant. *E*-value refers to the minimum association strength that unmeasured confounding factors must simultaneously possess with treatment group assignment and outcome variables to explain the observed point estimate or 95% CI lower limit as null [i.e., relative risk (RR) = 1.00] (15). Previous methodological studies indicate that when *E*-value > 1.5–2.0, it suggests that the observed association has strong resistance to interference.

## 3 Results

### 3.1 Comparison of clinical characteristics between survivor and non-survivor groups

This study enrolled a total of 133 pediatric HLH patients, who were divided into a survivor group (*n* = 104) and a non-survivor group (*n* = 29) based on their 30-day survival outcomes. There were no statistically significant differences between the two groups in terms of gender and age baseline indicators (*P* > 0.05). Regarding clinical manifestations and treatment, the non-survivor group exhibited significantly higher rates of CNS involvement (28% vs. 7%, *P* = 0.004) and imaging-confirmed hepatomegaly (97% vs. 69%, *P* = 0.006) compared to the survivor group, while the proportion receiving cyclosporine treatment was significantly lower than the survivor group (14% vs. 37%, *P* = 0.035). In terms of laboratory indicators, compared to the survivor group, the non-survivor group showed significantly elevated levels of CRP, PCT, RDW, APTT, and AST (*P* < 0.05), while MCH and TP levels were significantly decreased (*P* < 0.05) (Table 1).

**Table 1:** Comparison of clinical characteristics between survivor and non-survivor groups.

Items	Total (*n* = 133)	Survivor group (*n* = 104)	Non-survivor group (*n* = 29)	Statistical value	*P*
Demographic characteristics					
Sex (Female)	59 (44%)	48 (46%)	11 (38%)	*χ*^2^ = 0.333	0.564
Age (years)	2.25 (1.00, 5.00)	2.00 (1.00, 4.87)	3.00 (1.00, 6.00)	*Z* = −0.501	0.616
Clinical manifestations and treatment					
Cyclosporine treatment	38 (37%)	42 (32%)	4 (14%)	*χ*^2^ = 4.436	0.035
Hepatomegaly (imaging)	100 (75%)	72 (69%)	28 (97%)	*χ*^2^ = 7.669	0.006
CNS involvement	15 (11%)	7 (7%)	8 (28%)	*χ*^2^ = 8.274	0.004
Splenomegaly (imaging)	100 (75%)	77 (74%)	23 (79%)	*χ*^2^ = 0.114	0.735
Laboratory indicators					
NK cell activity	5.59 (3.49, 8.52)	5.38 (3.28, 7.58)	7.11 (4.03, 9.75)	*Z* = −1.895	0.058
CRP (mg/L)	23.63 (6.40, 70.42)	28.70 (7.50, 78.90)	43.30 (19.90, 124.00)	*Z* = −2.231	0.026
PCT (ng/mL)	1.10 (0.31, 3.69)	0.89 (0.25, 2.12)	1.89 (0.84, 5.22)	*Z* = −2.237	0.025
WBC (×10^9^/L)	4.45 (2.48, 8.26)	4.50 (2.51, 9.89)	7.22 (2.90, 11.20)	*Z* = −1.464	0.143
ANC (×10^9^/L)	1.33 (0.70, 2.37)	1.27 (0.70, 3.30)	2.45 (0.90, 6.27)	*Z* = −1.806	0.071
LYM (×10^9^/L)	2.14 (0.93, 4.05)	1.95 (0.94, 3.46)	2.78 (0.80, 4.88)	*Z* = −0.444	0.659
Monocytes(×10^9^/L)	0.43 (0.16, 0.85)	0.40 (0.16, 0.78)	0.50 (0.11, 1.47)	*Z* = −0.887	0.377
RBC (×10^12^/L)	3.56 ± 0.83	3.59 ± 0.86	3.44 ± 0.70	*t* = 0.984	0.327
Hb (g/L)	89.18 ± 18.63	90.80 ± 19.29	83.37 ± 20.80	*t* = 1.706	0.090
MCH (pg)	26.00 (24.00, 27.30)	26.40 (24.50, 27.60)	24.50 (22.30, 26.00)	*Z* = −3.076	0.002
MCHC (g/L)	329.61 ± 16.49	330.76 ± 16.70	334.88 ± 15.26	*t* = −1.594	0.114
RDW (%)	15.20 (13.90, 18.90)	15.05 (13.67, 17.83)	16.80 (14.20, 19.80)	*Z* = −2.198	0.028
PLT (×10^9^/L)	62.00 (35.00, 100.00)	66.00 (37.00, 100.25)	41.30 (23.00, 92.70)	*Z* = −1.814	0.070
Reticulocyte (%)	1.12 (0.50, 2.66)	1.12 (0.47, 2.72)	1.08 (0.80, 2.44)	*Z* = −0.038	0.970
FIB (g/L)	1.64 (1.09, 2.30)	1.67 (1.09, 2.40)	1.60 (1.09, 1.95)	*Z* = −0.733	0.464
APTT (s)	38.40 (30.70, 50.00)	36.05 (29.82, 44.90)	51.50 (41.00, 74.40)	*Z* = −4.070	<0.001
ALT (U/L)	131.50 (48.30, 313.20)	126.00 (39.70, 267.10)	159.50 (69.50, 344.00)	*Z* = −1.363	0.173
AST (U/L)	204.80 (67.40, 505.00)	183.40 (58.50, 373.83)	272.30 (129.20, 966.70)	*Z* = −2.109	0.035
LDH (U/L)	915.00 (494.00, 1, 826.00)	829.05 (479.72, 1,679.50)	1,076.90 (742.00, 2,949.00)	*Z* = −1.628	0.104
TP (g/L)	55.92 ± 10.56	56.74 ± 9.87	52.97 ± 12.47	*t* = 2.076	0.040
SF (µg/L)	1,808.00 (1,054.00, 10,195.00)	1,760.50(995.00, 9,093.00)	7,290.00(1,500.00, 20,923.00)	*Z* = −1.831	0.067

### 3.2 Screening of key prognostic factors and feature importance assessment

Univariate logistic regression analysis revealed that imaging-confirmed hepatomegaly (*OR* = 12.4444, 95% CI: 1.6219–95.4848, *P* = 0.015) and CNS involvement (*OR* = 5.2789, 95% CI: 1.7246–16.1584, *P* = 0.004) demonstrated extremely high predictive value for mortality risk. Additionally, among laboratory indicators, elevated APTT (*OR* = 1.0533, 95% CI: 1.0258–1.0816, *P* < 0.001), CRP (*OR* = 1.0110, 95% CI: 1.0022–1.0198, *P* = 0.014), and RDW (*OR* = 1.1181, 95% CI: 1.0021–1.2476, *P* = 0.046) were confirmed as independent risk factors for death. Cyclosporine treatment significantly reduced the mortality risk in children (*OR* = 0.2779, 95% CI: 0.0899–0.8588, *P* = 0.026). Meanwhile, higher MCH levels at admission also exhibited a protective effect (*OR* = 0.8560, 95% CI: 0.7590–0.9654, *P* = 0.011). To evaluate the robustness of the above results against potential unmeasured confounding factors, E-values were calculated. The results showed that the E-values for imaging-confirmed hepatomegaly and CNS involvement were as high as 24.3784 and 10.0316, respectively, suggesting that the strong associations of these two core variables with prognosis are difficult to be explained by potential unknown confounders (Table 2).

**Table 2:** Univariate logistic regression analysis of prognostic factors in children with HLH.

Items	*β*	*SE*	Wald*χ*2	*P*	*OR* (95% CI)	E-value for *OR*	E-value for
lower *CI* of *OR*							
Hepatomegaly (imaging)	2.521	1.040	5.881	0.015	12.4444 (1.6219–95.4848)	24.3784	2.6262
CNS involvement	1.664	0.571	8.496	0.004	5.2789 (1.7246–16.1584)	10.0316	2.8425
Cyclosporine treatment	−1.281	0.576	4.948	0.026	0.2779 (0.0899–0.8588)	6.6564	1.6019
MCH	−0.155	0.061	6.423	0.011	0.8560 (0.7590–0.9654)	1.6115	1.2286
RDW	0.112	0.056	3.990	0.046	1.1181 (1.0021–1.2476)	1.4816	1.0480
APTT	0.052	0.014	14.800	<0.001	1.0533 (1.0258–1.0816)	1.2903	1.1885
CRP	0.011	0.004	6.049	0.014	1.0110 (1.0022–1.0198)	1.1162	1.0493
PCT	0.008	0.011	0.525	0.469	1.0083 (0.9860–1.0311)	—	—
AST	<0.001	<0.001	0.909	0.340	1.0001 (0.9999–1.0004)	—	—
TP	−0.035	0.021	2.857	0.091	0.9653 (0.9265–1.0057)	—	—

**Figure 1:**
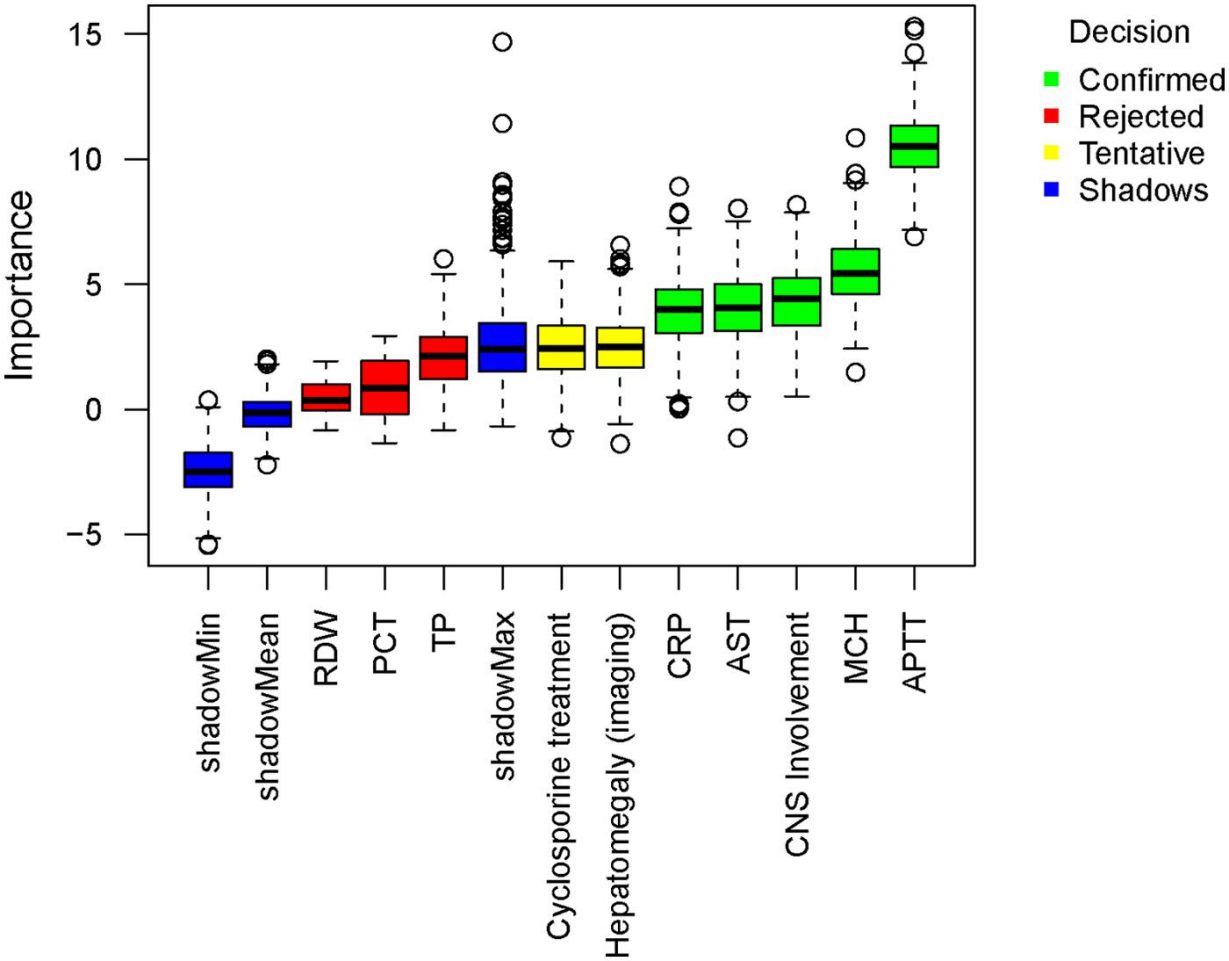
Feature importance ranking for predicting 30-day mortality in pediatric HLH patients based on the Boruta algorithm (Total *n* = 133). The algorithm was executed with 500 iterations. Boxplots represent the distribution of *Z*-scores for each candidate feature compared to their corresponding shadow features. Green boxes indicate confirmed key predictive features whose importance significantly exceeded the maximum *Z*-score of shadow features (Confidence level *P* < 0.01), whereas red boxes denote rejected features with no significant predictive value.

Based on the preliminary univariate exploration and clinical relevance, a complete list of 10 candidate variables was subsequently fed into the Boruta algorithm for core feature selection. These candidates included: imaging-confirmed hepatomegaly, CNS involvement, cyclosporine treatment, mean corpuscular hemoglobin (MCH), red cell distribution width (RDW), activated partial thromboplastin time (APTT), C-reactive protein (CRP), procalcitonin (PCT), aspartate aminotransferase (AST), and total protein (TP). To further precisely identify core features with the greatest predictive value, the Boruta algorithm was employed for secondary screening of candidate variables. The algorithm compares the *Z*-scores of original features with shadow features. Results showed that APTT had the highest mean *Z*-score (Mean *Z*-Score = 10.522), indicating its greatest predictive contribution, followed by MCH (5.527), CNS involvement (4.369), AST (4.080), and CRP (3.914), all significantly higher than the maximum *Z*-score of shadow features. Combining the Boruta algorithm and univariate logistic regression, six confirmed variables were finally determined for inclusion in subsequent analyses: APTT, MCH, CNS involvement, CRP, hepatomegaly, and cyclosporine treatment (Table 3 and Figure 1).

**Table 3:** Feature selection and importance ranking based on the boruta algorithm.

Items	Mean *Z*-Score	Median *Z*-Score	Min *Z*-Score	Max *Z*-Score	Decision
APTT	10.522	10.511	6.902	15.000	Confirmed
MCH	5.527	5.444	1.477	11.000	Confirmed
CNS involvement	4.369	4.429	0.515	8.000	Confirmed
AST	4.080	4.057	−1.145	8.000	Confirmed
CRP	3.914	3.980	0.016	9.000	Confirmed
Hepatomegaly (imaging)	2.513	2.509	−1.380	7.000	Confirmed
Cyclosporine treatment	2.453	2.431	−1.132	6.000	Confirmed
Total protein (TP)	2.130	2.121	−0.839	6.000	Rejected
PCT	0.764	0.834	−1.349	3.000	Rejected
RDW	0.537	0.351	−0.834	2.000	Rejected

### 3.3 Multicollinearity diagnostics of variables

Multicollinearity diagnostics were performed on the key features screened by univariate logistic regression and the Boruta algorithm. Results showed that the GVIF for CNS involvement, CRP, MCH, APTT, cyclosporine treatment, and imaging-confirmed hepatomegaly ranged from 1.011 to 1.175, with corresponding tolerance values all greater than 0.85, demonstrating good stability (Table 4).

**Table 4:** Collinearity diagnostics of the variables.

Items	GVIF	Tolerance (1/GVIF)
CNS involvement	1.173	0.853
CRP	1.026	0.975
MCH	1.072	0.933
APTT	1.175	0.851
Cyclosporine treatment	1.011	0.989
Hepatomegaly (imaging)	1.014	0.987

### 3.4 Fitting of LCA models

To identify potential clinical phenotypes within the pediatric HLH population and determine the optimal number of classes, LCA was applied to the data screened by univariate logistic regression and the Boruta algorithm. Comprehensive comparison of AIC, BIC, and aBIC revealed that Model 2 had the lowest values across all metrics (AIC = 1,507.750, BIC = 1,573.030, aBIC = 1,493.840). Although the 3-class model demonstrated a higher entropy (0.828), we prioritized information criteria, particularly BIC, over entropy for final model selection. BIC is widely recognized as a more robust metric for determining the true number of latent classes because it rigorously penalizes model complexity, thereby preventing overfitting—a crucial consideration given our specific sample size (16). Consequently, Model 2 was determined to indicate the best model fit, with a class probability distribution of 0.28 and 0.72 (Table 5).

**Table 5:** Model fit indices for latent class analysis.

Model	AIC	BIC	aBIC	P_LMR	P_BLRT	Entropy	Probability
1	1,538.350	1,587.350	1,535.070	—	—	—	—
2	1,507.750	1,573.030	1,493.840	<0.001	<0.001	0.702	0.28/0.72
3	1,508.990	1,618.820	1,498.620	0.002	0.040	0.828	0.16/0.67/0.17
4	1,515.090	1,655.160	1,508.270	0.012	0.220	0.792	0.35/0.12/0.35/0.18
5	1,512.230	1,697.220	1,494.780	0.063	0.400	0.869	0.18/0.17/0.10/0.45/0.11

To elucidate the specific clinical profiles of the identified subtypes, the conditional response probabilities for categorical indicators and the estimated means for continuous indicators across the two latent classes were evaluated. Compared to the low-risk group (Class 1), the high-risk group (Class 2) was characterized by a distinct pattern of severe systemic dysregulation and coagulation dysfunction. Specifically, patients in the high-risk class exhibited a substantially higher estimated mean of APTT (67.72 ± 28.62 vs. 34.85 ± 7.57 s) and elevated CRP levels (48.83 ± 44.08 vs. 44.95 ± 46.48 mg/L). For categorical variables, the high-risk group demonstrated a remarkably higher probability of imaging-confirmed hepatomegaly (91.89% vs. 68.75%). Notably, the conditional probability of CNS involvement in the low-risk group was exactly zero (0.00% vs. 40.54% in the high-risk group) (Table 6).

**Table 6:** Conditional response probabilities and estimated means of the 6 core variables across the two latent classes.

Items	Low-risk Group (72%)	High-risk Group (28%)
CNS involvement	0.000 (0.00%)	0.405 (40.54%)
Hepatomegaly (imaging)	0.688 (68.75%)	0.919 (91.89%)
Cyclosporine treatment	0.292 (29.17%)	0.378 (37.84%)
APTT (s)	34.85 ± 7.57	67.72 ± 28.62
MCH (pg)	25.00 ± 3.35	25.52 ± 3.16
CRP (mg/L)	44.95 ± 46.48	48.83 ± 44.08

### 3.5 Clinical outcome analysis based on LCA

**Figure 2:**
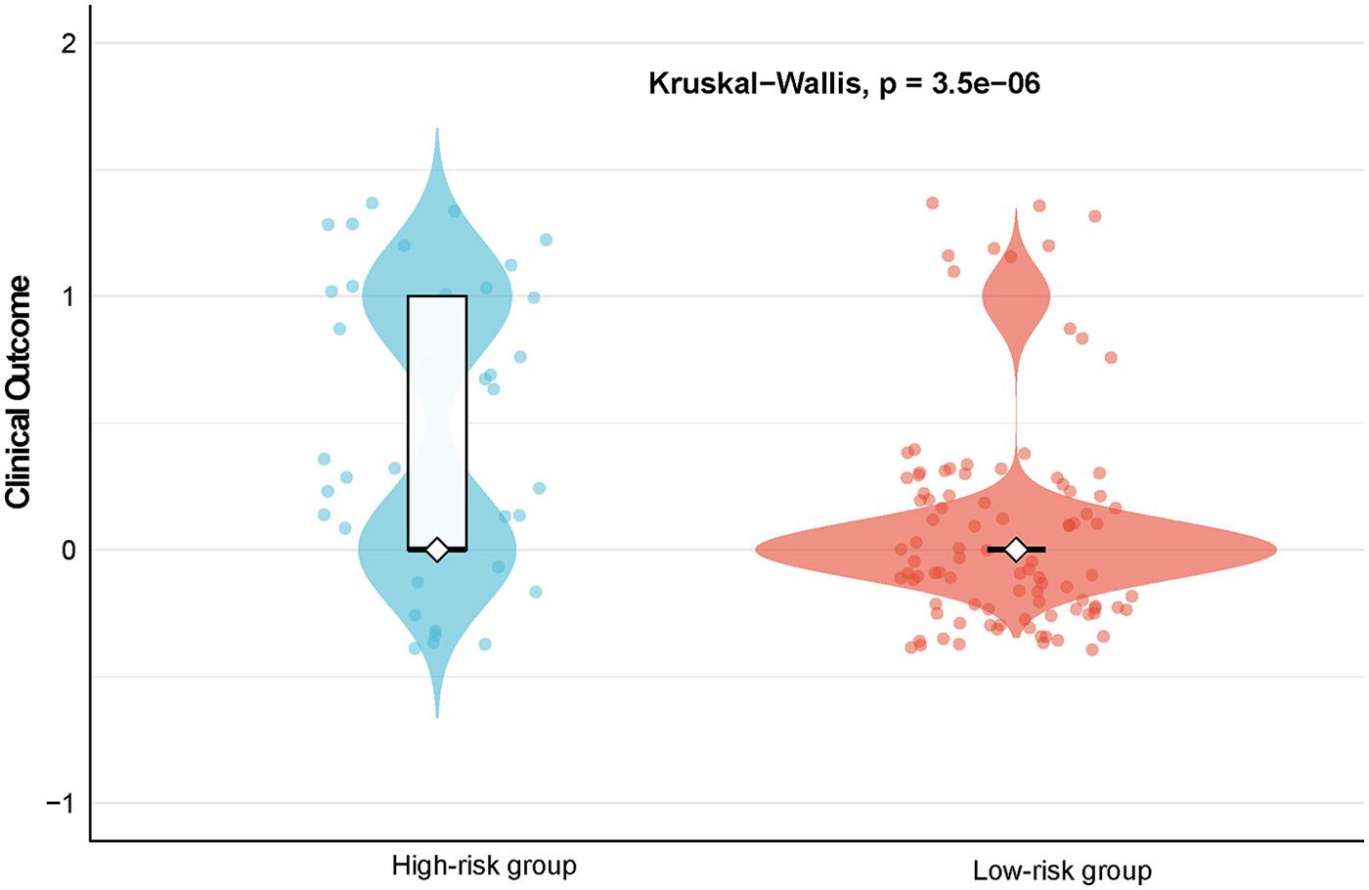
Violin plot illustrating the distribution of clinical outcomes across the identified latent classes (total *n* = 133). The width of the violin represents the probability density of the data at different outcome values. Differences in clinical outcome distributions between the low-risk and high-risk groups were evaluated using the non-parametric Kruskal–Wallis test. The result reveals a highly significant distinction in mortality trajectories between the two subgroups (*P* = 3.5 × 10⁻⁶).

To validate the clinical significance of the subtypes identified by LCA in terms of prognosis, this study further analyzed the distribution differences in clinical outcomes between different latent class groups. The two subgroups determined by the LCA model exhibited distinctly different prognostic characteristics and were defined as low-risk group and high-risk group. Non-parametric Kruskal–Wallis test analysis revealed statistically significant differences in clinical outcomes between the two groups (*P* = 3.5 × 10^−6^), indicating that the LCA model constructed based on key peripheral blood indicators can effectively identify pediatric HLH subgroups with different mortality risk trajectories (Figure 2).

**Figure 3:**
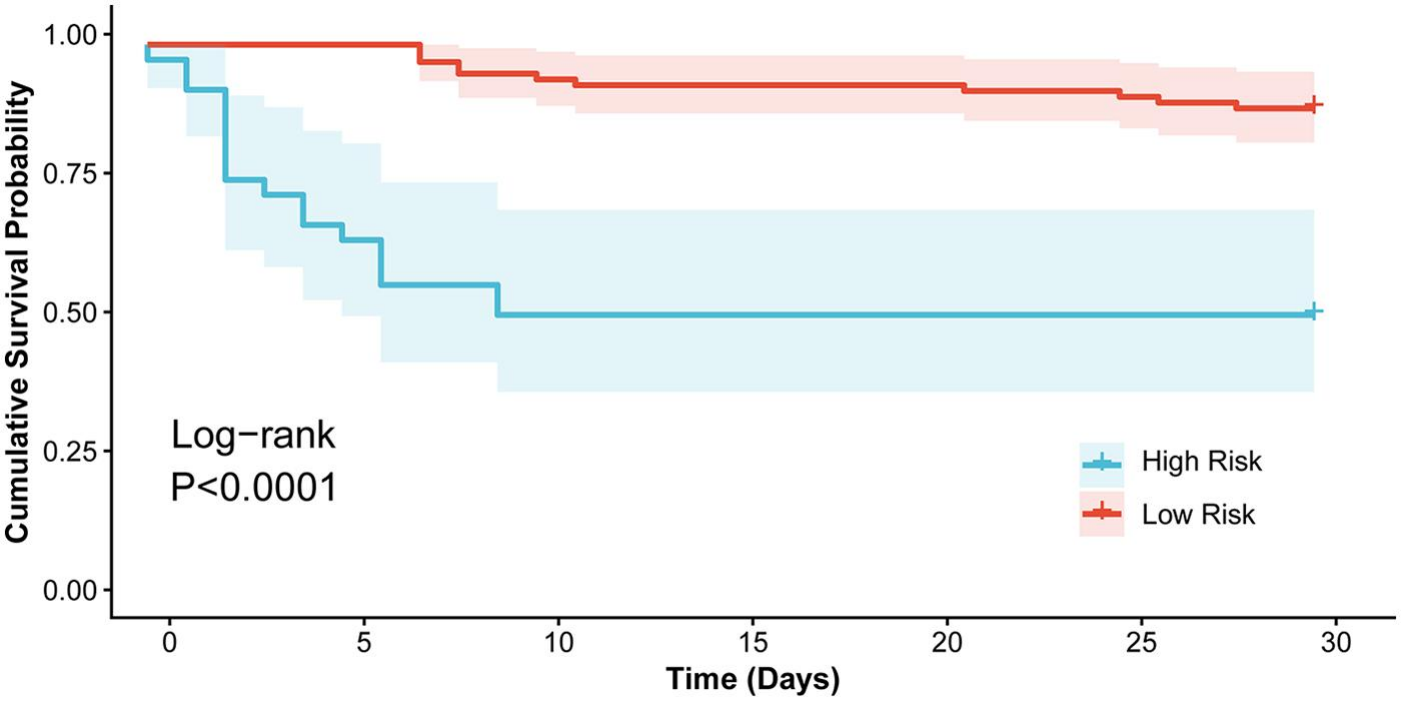
Kaplan–Meier survival curves for 30-day mortality in pediatric HLH patients stratified by latent classes (Total *n* = 133; low-risk group, 72%; high-risk group, 28%). Group differences in cumulative survival probabilities were statistically assessed using the Log-rank test. The survival analysis demonstrates a significantly lower cumulative survival rate and a rapid clinical deterioration trajectory in the high-risk group compared to the low-risk group (Log-rank *P* < 0.001).

To validate the clinical significance of the subtypes identified by LCA in terms of prognosis, Cox proportional hazards regression and Kaplan–Meier survival analysis were employed. The two subgroups exhibited distinctly different prognostic trajectories and were defined as the low-risk group and high-risk group. Univariate Cox regression analysis revealed that patients in the high-risk group faced a 7.4184-fold increased risk of 30-day mortality compared to those in the low-risk group (*HR* = 7.4184, 95% CI: 2.6274–20.9205, *P* < 0.001). Moreover, Kaplan–Meier analysis visually and statistically supported these findings, demonstrating that the 30-day cumulative survival rate was significantly lower in the high-risk group (51.40% vs. 88.50%, Log-rank *P* < 0.001). Patients in the high-risk group exhibited rapid clinical deterioration with a median survival time of only 2.50 days (95% CI: 2.00–6.00), whereas the median survival time was not reached in the low-risk group (Figure 3).

### 3.6 Importance scoring and SHAP model interpretability analysis of key predictive features

**Figure 4:**
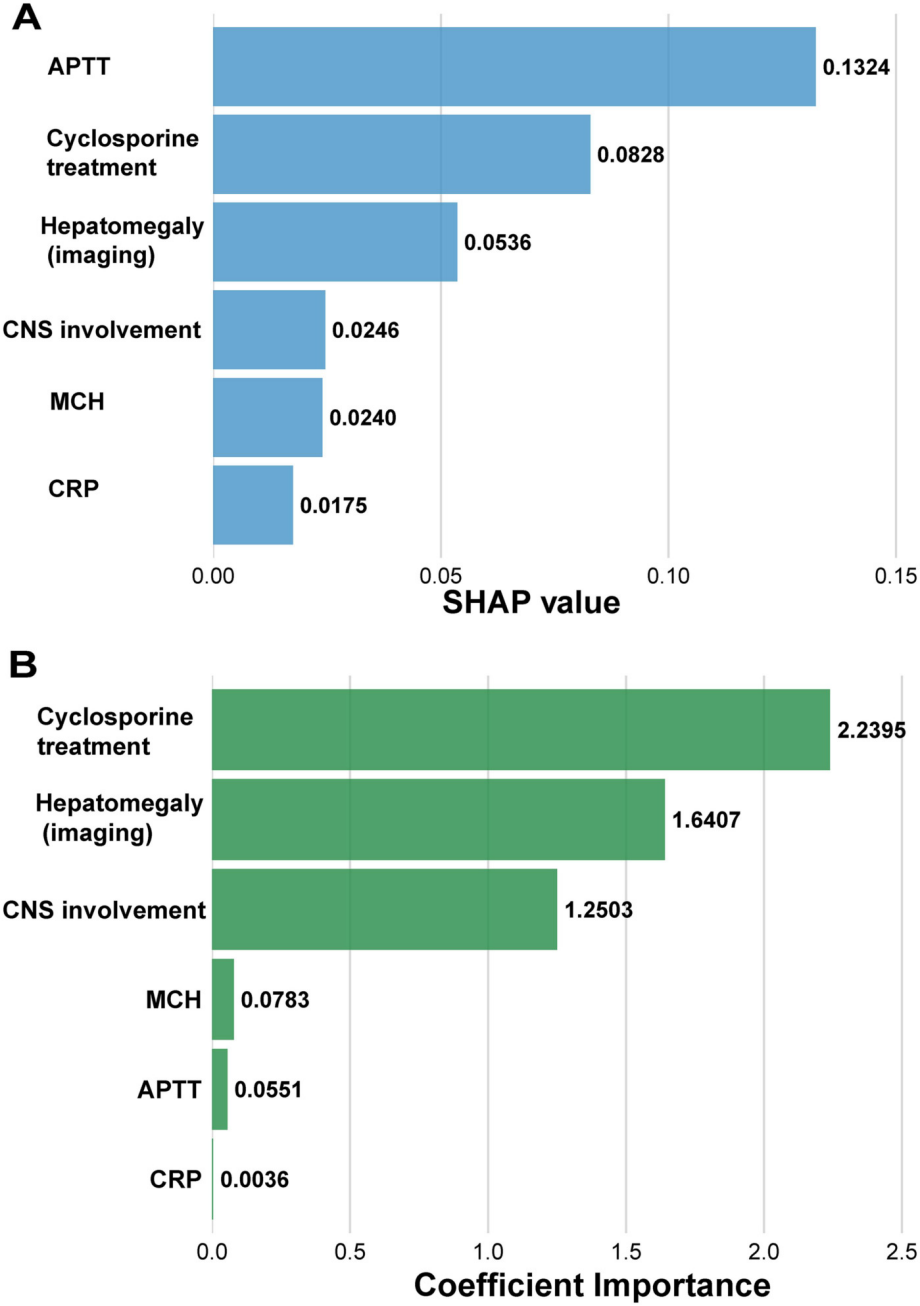
Interpretability analysis of the core predictive features (Total *n* = 133). **(A)** SHAP summary plot. Each dot represents a single patient, with colors indicating the original feature value (red for high, blue for low) and the horizontal axis quantifying the positive or negative impact on the model's 30-day mortality prediction. **(B)** Variable importance ranking based on model coefficients, highlighting the absolute relative contribution weight of each feature to the final risk stratification.

This study employed two methods—coefficient importance and SHAP values—to evaluate the predictive contribution of variables. Coefficient importance analysis showed that cyclosporine treatment, imaging-confirmed hepatomegaly, and CNS involvement had the highest predictive contributions. SHAP analysis revealed that APTT had the highest predictive contribution, followed by cyclosporine treatment, imaging-confirmed hepatomegaly, CNS involvement, MCH, and CRP (Figures 4A,B).

### 3.7 Dose-response relationship analysis of continuous variables based on RCS models

**Figure 5:**
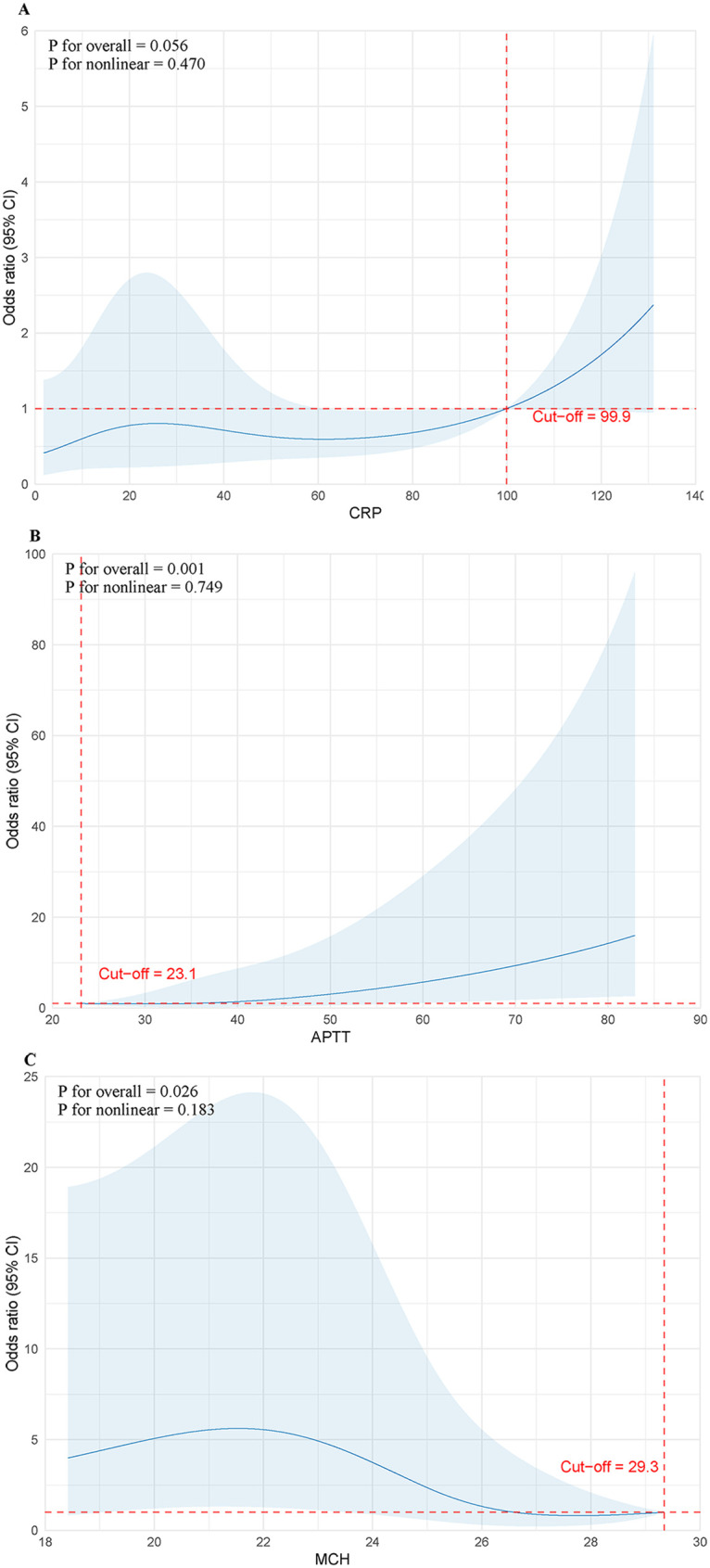
Dose-response relationships between continuous peripheral blood indicators and 30-day mortality risk modeled using RCS (Total *n* = 133). **(A)** Association between CRP and mortality risk; **(B)** Association between APTT and mortality risk (Overall statistical association *P* = 0.001); **(C)** Association between MCH and mortality risk (Overall statistical association *P* = 0.026). Solid blue lines represent the estimated OR, and shaded areas represent the 95% CI. Red dashed lines indicate the reference baseline (OR = 1.0) and the precise optimal clinical cut-off values (CRP = 99.9 mg/L; APTT = 23.1 s; MCH = 29.3 pg) which were mathematically determined via piecewise linear regression.

To further refine the analysis of specific association patterns between key continuous variables and 30-day mortality risk in children, RCS models were constructed to explore potential dose-response relationships. Analysis results showed that APTT exhibited a highly significant statistical association with mortality risk $\left(P_{\mathrm{overall}}=0.001\right)$, presenting an overall monotonically increasing trend. MCH demonstrated a significant protective trend $\left(P_{\mathrm{overall}}=0.026\right)$, with mortality risk gradually decreasing as MCH levels increased (Figures 5A–C).

## 4 Discussion

Although the popularization of salvage therapies such as HSCT has significantly improved the long-term survival prospects of pediatric HLH, this has not completely resolved the clinical dilemma of high mortality rates in the early period (30 days) following diagnosis. During this critical therapeutic window, due to the lack of assessment tools capable of integrating multidimensional clinical information, some children die from disease deterioration before induction therapy takes effect. The primary incremental value of this study lies in addressing the intrinsic limitations of conventional prognostic approaches to offer a comprehensive risk stratification framework. Existing HLH prognostic studies predominantly utilize traditional linear regression or single machine learning techniques, which fundamentally operate on a variable-centric paradigm. These methods typically assume that risk factors exert uniform linear effects across the entire patient population, making it difficult to capture the extreme phenotypic heterogeneity and complex, nonlinear interactions inherent to the acute cytokine storm of HLH. To overcome this, our study established a synergistic, patient-oriented analytical strategy. The Boruta algorithm was uniquely suited for handling the highly fluctuating inflammatory network of HLH, effectively filtering out high-dimensional background noise and isolating genuine core predictors of mortality without human bias. Subsequently, feeding these purified features into the LCA model enabled us to move beyond relying on single isolated thresholds. This integration effectively parsed a seemingly uniform pediatric population into hidden sub-populations, distinctly identifying a high-risk phenotype defined not merely by solitary abnormal values, but by a catastrophic cluster of concurrent physiological breakdowns. Based on this methodology, we compared our LCA-derived findings with a recently published prediction model for elderly HLH (17) at the pathological mechanism level, revealing significant age-specific differences: mortality risk in elderly patients is primarily associated with indicators reflecting renal dysfunction and metabolic burden (such as urea nitrogen), suggesting their prognosis is limited by declining organ functional reserve; whereas our model clarifies that early mortality in the highly heterogeneous pediatric population is intimately related to an explosive phenotype dominated by uncontrolled coagulation cascade reactions (APTT) and severe multi-organ infiltration (CNS involvement).

This study found that APTT occupies the highest predictive weight in the LCA model, and its nonlinear impact on prognosis profoundly reveals the unique coagulation pathological mechanism of pediatric HLH. The surge in cytokines induced by HLH [such as interleukin-6 (IL-6) and tumor necrosis factor-alpha (TNF-α)] can induce abnormal expression of tissue factor on the surface of monocytes and endothelial cells, thereby activating the coagulation cascade; meanwhile, the pediatric coagulation system is still in the developmental stage with weak physiological compensatory capacity for such explosive activation, making it extremely susceptible to rapid consumption of endogenous coagulation factors (such as factors VIII and IX) and anticoagulant proteins, ultimately triggering the disseminated intravascular coagulation process and exacerbating systemic microcirculatory disturbance (18). Regarding the RCS-derived threshold statistically determined via segmented regression, the cut-off for APTT (where OR=1.0) was observed at a relatively low value of 23.1 s compared to conventional clinical upper limits. It is crucial to clarify that this spline-derived threshold does not represent a universal clinical diagnostic boundary for coagulopathy, but rather indicates the statistical baseline of lowest mortality risk dictated by the specific pathophysiological mechanisms of pediatric HLH. The monotonic increase in risk from this low baseline suggests that under the unique hyperinflammatory and cytokine storm conditions of pediatric HLH, even upward fluctuations within the normal clinical range of APTT may signal the insidious onset of rapid coagulation factor consumption.

Coexisting with coagulation disorders is CNS involvement, a key prognostic feature. Previous research on sepsis-associated encephalopathy has confirmed that neurological symptoms are often early warning signals of uncontrolled systemic inflammatory response (19); in HLH, CNS involvement indicates that the intensity of the inflammatory storm has breached the physiological defense limits of the blood-brain barrier (20). This barrier breakdown exposes the central nervous system to direct attack by systemic inflammatory mediators, allowing high concentrations of interferon- (IFN-*γ*) and CXCL9 chemokine to penetrate into the brain (21), not only directly mediating the activation and damage of neuroglial cells but also suggesting that peripherally activated lymphocytes have undergone multi-organ infiltration and migration (22, 23). Therefore, the high weight of CNS involvement in the model does not merely indicate focal damage to brain parenchyma but rather predicts that children are in an extremely active systemic inflammatory state prone to multi-organ failure, which may explain why CNS involvement frequently precedes circulatory failure and signals adverse prognosis.

Additionally, this study identified decreased MCH as a predictor. Unlike anemia caused by acute blood loss, the hypochromic microcytic anemia characteristics presented in pediatric HLH are attributed to inflammation-induced iron metabolism reprogramming mechanisms. Sustained high inflammatory states stimulate the liver to upregulate hepcidin expression, which blocks iron release from the reticuloendothelial system by degrading membrane iron transport proteins, leading to functional iron restriction in the erythroid hematopoietic system despite adequate iron stores (24). Since changes in red blood cell morphology require a relatively long metabolic cycle, decreased MCH suggests that children have suffered sustained immune attack and physiological reserve depletion during the latent period before diagnosis. This chronic physiological exhaustion state of decreased MCH intertwines with widespread organ infiltration indicated by hepatomegaly and acute inflammatory burden suggested by CRP surge, jointly constituting a multiplicative effect on mortality risk (25). Similarly, the RCS-derived reference point for MCH exceeds the median values of our cohort. This finding is biologically consistent rather than a sign of threshold instability, as it is deeply rooted in the disease-specific context of pediatric HLH. Because most pediatric HLH patients already present with varying degrees of HLH-driven, inflammation-induced iron restriction at diagnosis, the median cohort values inherently reflect this unique pathological state. The higher RCS threshold simply demarcates the optimal physiological baseline where mortality risk normalizes, emphasizing that any deviation below this ideal range—even if common among the cohort—carries an incrementally increased risk. Correspondingly, a specific tipping point was mathematically identified for CRP at 99.9 mg/L, beyond which the mortality risk escalated sharply, marking the critical boundary of irreversible systemic inflammatory damage. Therapeutically, cyclosporine binds with intracellular cyclophilin to form a complex that specifically inhibits the phosphatase activity of calcineurin, thereby blocking dephosphorylation of nuclear factor of activated T cells and its translocation into the nucleus (26). This key step directly inhibits transcription of pro-inflammatory cytokine genes including interleukin-2 and IFN-*γ*, thus restraining clonal expansion of cytotoxic T lymphocytes and sustained activation of macrophages (27). However, because cyclosporine administration reflects early therapeutic decision-making rather than baseline biological status, residual confounding and treatment indication bias cannot be excluded.

In summary, this study constructed a 30-day mortality risk stratification model for pediatric HLH by deploying a combined Boruta and LCA analytical strategy based on routine peripheral blood indicators. By shifting from traditional linear estimation to unsupervised phenotypic clustering, this approach demonstrated distinct advantages over prior methods in capturing population heterogeneity. The model not only confirmed the critical relevance of APTT, CNS involvement, and MCH in the immune-coagulation pathological cascade reaction but also quantified early diagnostic mortality risk by effectively identifying a clinically divergent high-risk phenotype. However, this study still has certain limitations that must be comprehensively acknowledged. First, inherent to its retrospective single-center design, this study is susceptible to specific selection and information biases. Regarding selection bias, our cohort was derived from a single tertiary pediatric medical center over a nearly 10-year span. Patients referred to our facility might represent cases with higher baseline severity or specific referral patterns, limiting the broad generalizability of our latent classes to primary care or different demographic settings. Additionally, excluding patients with >20% missing core data could inadvertently introduce survivorship bias if those excluded were the most critically ill patients who deteriorated or died before complete laboratory panels could be drawn. Regarding information bias, retrospective data extraction relies heavily on the fidelity of historical electronic medical records. Although we strictly limited the data collection window to within 24 h before induction therapy to minimize treatment interference, the lack of standardized prospective protocols means that unmeasured temporal variations in laboratory assays over the years, as well as the inevitable missingness of specialized immune markers (such as sCD25 and cytokine profiles) due to non-routine clinical detection, may have led to the omission of critical biological information. Additionally, the impact of the limited number of death events (*n* = 29) on the stability of the LCA classification warrants in-depth consideration. Although our total sample size (*n* = 133) mathematically satisfied the minimum threshold for latent class enumeration, the variables inputted into the LCA model were initially screened against these 29 terminal events. This relatively low events per variable (EPV) ratio inherently increases the risk of statistical overfitting during the preliminary Boruta feature selection phase. Consequently, this constraint may impact the robustness of the downstream LCA; slight variations in a larger or different cohort could potentially shift the estimated class boundaries, latent proportions, or conditional probabilities. Therefore, while the identified high-risk phenotype exhibits strong clinical and pathophysiological coherence, the statistical fragility induced by the limited death events means our current latent classes must be interpreted as a preliminary exploratory framework rather than definitive diagnostic criteria. Beyond these data constraints, it is essential to emphasize that LCA is fundamentally a descriptive stratification technique rather than a tool with an independent predictive function. Unlike clinical scoring systems or regression equations that can directly calculate individualized risk probabilities for new, isolated patients, LCA clusters individuals based on the specific underlying covariance structure of the given dataset. Thus, it cannot be directly applied to independently predict outcomes for a single new patient without integrating them into a broader population matrix. Finally, the current model has not yet been validated in external independent cohorts due to significant methodological barriers. External validation of an LCA mixture model requires sharing and harmonizing complete, high-dimensional raw datasets across different institutions to reconstruct the latent distributions. Given the rarity of pediatric HLH and the current absence of standardized data-sharing protocols among regional medical centers, conducting a rigorous retrospective external validation was not feasible. Future research intends to conduct multicenter, large-sample prospective cohort studies and attempt to incorporate multi-dimensional omics features for external validation to mitigate these inherent biases, enhance the model's robustness and clinical applicability, and provide more solid evidence-based support for precision stratified diagnosis and treatment of pediatric HLH.

## 5 Conclusion

The integration of the Boruta feature selection algorithm with LCA provides a robust analytical strategy for identifying clinical subtypes of pediatric HLH with distinct mortality risk trajectories based on routine peripheral blood indices. Our study successfully stratified patients into low-risk and high-risk groups, revealing statistically significant differences in 30-day clinical outcomes. Among the identified key predictors, APTT demonstrated the highest predictive contribution, exhibiting a monotonically increasing relationship with mortality risk, whereas elevated MCH levels showed a significant protective trend. Additionally, CNS involvement and hepatomegaly were confirmed as critical indicators of poor prognosis. This combined prediction model effectively captures the early signals of immune-coagulation cascade dysregulation and systemic inflammation, offering predictive efficacy and clinical value for early risk stratification and personalized intervention in pediatric HLH patients.

## Data Availability

The raw data supporting the conclusions of this article will be made available by the authors, without undue reservation.
